# Efficacy and Safety of Focal and Multifocal Cryoballoon Ablation for Barrett’s Esophagus: A Systematic Review and Meta-analysis

**DOI:** 10.1055/a-2951-0511

**Published:** 2026-09-14

**Authors:** Mohammad Al Hayek, Bisher Sawaf, Davide Massimi, Marco Spadaccini, Obada Tarabichi, Mohamedhen Vall Nounou, Muhammed Elhadi, Cesare Hassan, Mohammad Al-Haddad

**Affiliations:** 1 Faculty of Medicine 108042 Damascus UniversityDamascusDamascus GovernorateSyrian Arab Republic; 2 Internal Medicine 7923 The University of ToledoToledoOhioUnited States; 3 Department of Gastroenterology 551905 IRCCS Humanitas Research HospitalRozzanoLombardiaItaly; 4 Endoscopy Unit, Gastroenterology Department 9268 IRCCS Humanitas Research HospitalRozzanoLombardiaItaly; 5 Department of Biomedical Sciences 437807 Humanitas UniversityMilanLombardyItaly; 6 College of Medicine 59105 University of SharjahSharjahSharjahUnited Arab Emirates; 7 Faculty of Medicine 107915 University of NouakchottNouakchottNouakchottMauritania; 8 Faculty of Medicine 125562 University of TripoliTripoli DistrictTripoliLibya; 9 College of Medicine 34973 Korea UniversitySeongbuk-guSeoulKorea (the Republic of); 10 Division of GastroenterologyIndiana University School of MedicineIndianapolisIndianaUnited States; 11 Division of Gastroenterology and Hepatology, School of Medicine 54658 The University of JordanAmmanAmman GovernorateJordan

**Keywords:** Barrett’s esophagus, cryoballoon ablation, endoscopic eradication therapy, cryotherapy, intestinal metaplasia, esophageal dysplasia

## Abstract

**Background and Study Aims** Cryoballoon ablation has emerged as a novel modality for the treatment of Barrett’s esophagus (BE), but comprehensive evaluation of its efficacy and safety remains limited. We performed a systematic review and meta-analysis to assess the outcomes of focal and multifocal cryoballoon ablation.

**Methods** A systematic search of the PubMed, Embase, Web of Science, and Cochrane Library databases was conducted through March 10, 2026. Studies evaluating cryoballoon ablation in adult patients with BE were included. The primary outcomes were complete eradication of intestinal metaplasia (CE-IM) and dysplasia (CE-D). Secondary outcomes included technical success and adverse events. A random-effects model was used.

**Results** Five prospective studies involving 337 patients were analyzed. Pooled CE-IM and CE-D rates were 84% (95%CI, 72–91%; *I* ^2^ = 84.5%) and 90% (95%CI 76–96%; *I* ^2^ = 88%) in intention-to-treat analyses, increasing to 92% (95%CI 87–96%; *I* ^2^ = 55.1%) and 98% (95%CI 94–99%; *I* ^2^ = 0%) in per-protocol analyses. Technical success was 96% (95%CI 94–98%; *I* ^2^ = 52.5%). The overall stricture rate was 6% (95% CI 2–18%; *I* ^2^ = 0%), with higher rates in multifocal ablation (12%) than in focal ablation (0%). Buried Barrett’s glands were rare (1%).

**Conclusions** Cryoballoon ablation is associated with high rates of CE-IM and CE-D and a favorable safety profile in patients with BE. However, the available evidence is limited to a small number of nonrandomized studies that predominantly included short-segment, noncircumferential BE and prior endoscopic mucosal resection and were associated with substantial heterogeneity. Larger, well-designed randomized trials with longer follow-up are needed to more definitively establish its comparative effectiveness and safety relative to established ablative modalities.

## Introduction

Barrett’s esophagus (BE) is a premalignant condition that can progress from intestinal metaplasia (IM) to low-grade dysplasia (LGD), high-grade dysplasia (HGD), and ultimately esophageal adenocarcinoma. 1 Current guidelines recommend endoscopic eradication therapy for BE with early neoplasia, consisting of endoscopic resection of visible lesions followed by ablation of residual Barrett’s mucosa. 2 3

Radiofrequency ablation (RFA) is the most established ablative modality and has demonstrated high efficacy. 4 5 6 However, its use is associated with adverse events, including esophageal strictures in 7%–15% of patients, 4 5 6 7 as well as postprocedural pain and dysphagia. 8 Other ablative techniques, including carbon dioxide cryotherapy, 9 liquid nitrogen spray cryotherapy, 10 11 12 and argon plasma coagulation (APC), 13 14 15 have been evaluated as alternative or adjunctive therapies.

Cryoballoon ablation has emerged as a promising modality, 16 offering improved tolerability compared with RFA 17 and the potential to preserve extracellular matrix integrity, which may facilitate deeper tissue ablation. 18 Focal cryoballoon ablation, designed to treat areas of approximately 2 cm ^2^ , has demonstrated favorable efficacy and safety in patients with short-segment BE. 19 20 21 More recently, multiple focal applications have been used for longer segments with encouraging results. 22 23 24

A retrospective study showed lower postprocedural pain and reduced analgesic requirements with cryoballoon ablation compared with RFA, while stricture rates were comparable. 25 Furthermore, a comparative retrospective study demonstrated that rates of BE recurrence and dysplastic recurrence were similar between RFA and cryoballoon ablation. 26 However, a comprehensive meta-analysis evaluating the efficacy and safety of focal and multifocal cryoballoon ablation is lacking. This study aimed to address this gap.

## Materials and methods

### Study Design and Registration

This systematic review and meta-analysis were conducted in accordance with the Preferred Reporting Items for Systematic Reviews and Meta-Analyses guidelines 27 28 ( **Supplementary Table S1** ). The study protocol was prospectively registered in the International Prospective Register of Systematic Reviews (PROSPERO; CRD420261366918).

### Search Strategy and Eligibility Criteria

A comprehensive literature search was performed from database inception through March 10, 2026, across PubMed, Embase, Web of Science, and the Cochrane Library. The search strategy incorporated controlled vocabulary and free-text terms related to “BE” and “cryoballoon ablation.” The full search strategy is provided in **Supplementary Table S2.**

Eligible studies included randomized and nonrandomized studies (prospective or retrospective) evaluating the technical success and safety of focal or multifocal cryoballoon ablation in adult patients (≥18 years) with BE. Studies were excluded if they involved pediatric populations, overlapping cohorts, conference abstracts, case reports, and case series including fewer than 10 patients, animal studies, or in vitro experiments. No restrictions were applied regarding language, geographic location, or publication date.

### Study Selection and Data Extraction

Study selection was performed in two stages: (1) screening of titles and abstracts and (2) full-text review. Two reviewers independently assessed all records at each stage. Discrepancies were resolved through discussion, with adjudication by a third reviewer when necessary.

Data were independently extracted by two reviewers (BS, OT) using a standardized data collection form. Extracted variables included study characteristics (first author, year, country, and sample size) and baseline patient characteristics (age, sex, body mass index, Prague classification (circumferential [C] and maximal [M] lengths), prior endoscopic mucosal resection (EMR), prior RFA, presence of hiatal hernia, prior fundoplication, and baseline histology). Outcome data were extracted separately. All data required for the predefined analyses were available in the published reports; therefore, no contact with the study authors for additional or missing data was necessary.

### Outcomes

The primary outcomes were complete eradication of IM (CE-IM) and CE of dysplasia (CE-D), each evaluated using both intention-to-treat and per-protocol analyses.

Secondary outcomes included technical success, defined as successful completion of the ablation procedure with delivery of therapy to the intended Barrett’s epithelium without device malfunction, premature termination, or inadequate targeting of the treatment area. Additional secondary outcomes included ablation time, total procedure time, esophageal stricture rate, and the incidence of buried Barrett’s glands.

A prespecified subgroup analysis was performed in patients without prior endoscopic ablation, assessing CE-IM and CE-D rates in both intention-to-treat and per-protocol populations.

### Risk of Bias Assessment

Risk of bias was independently assessed by two reviewers using the Risk Of Bias In Nonrandomized Studies of Interventions tool. 29 This instrument evaluates bias across seven domains: confounding, participant selection, classification of interventions, deviations from intended interventions, missing data, measurement of outcomes, and selection of reported results. Each domain was judged to have a low, moderate, serious, or critical risk of bias. Discrepancies were resolved through discussion, with adjudication by a third reviewer when necessary.

### Statistical Analysis

Statistical analyses were performed using RStudio (meta package). A random-effects model was used to account for both within-study and between-study variability. Pooled proportions for dichotomous outcomes and pooled means for continuous outcomes were calculated with corresponding 95% confidence intervals (CIs).

Sensitivity analyses were conducted using a leave-one-out approach to assess the robustness of the pooled estimates. Intention-to-treat analyses included all enrolled patients, whereas per-protocol analyses included only those who completed follow-up endoscopic assessments for outcome determination.

Statistical heterogeneity was assessed using Cochran’s Q (χ ^2^ ) test, the *I* ^2^ statistic, and the between-study variance (τ ^2^ ). Degrees of freedom (df) were calculated as the number of studies minus one. *I* ^2^ values greater than 50% were considered indicative of substantial heterogeneity. 30 The statistical significance of the pooled estimates was assessed using *Z* -tests for the overall effect, and the corresponding *p* values are reported in the forest plots. All statistical tests were two-sided, and *p* values < 0.05 were considered statistically significant.

Assessment of publication bias was planned for outcomes including at least 10 studies; however, this analysis was not performed because only five studies met the inclusion criteria. 31

## Results

### Studies Characteristics

The literature search identified 367 records. After removal of duplicates and screening of titles and abstracts, 11 articles underwent full-text review. Of these, five studies met the inclusion criteria and were included in the final analysis ( Fig. 1 ).

**Fig. 1:**
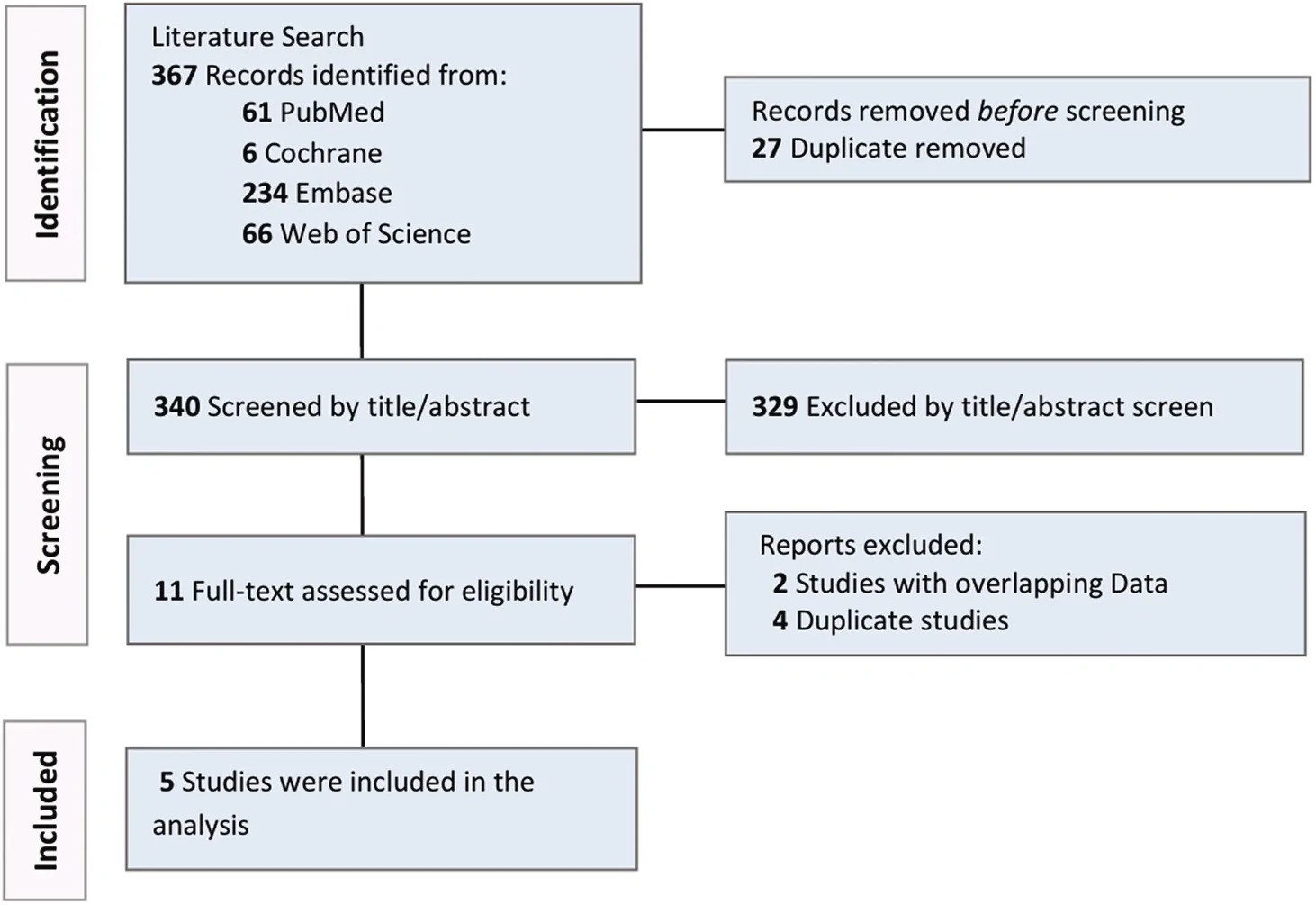
Preferred Reporting Items for Systematic Reviews and Meta-Analyses (PRISMA) 2020 flow diagram of study selection for the systematic review and meta-analysis of focal and multifocal cryoballoon ablation for Barrett’s esophagus. 27 28

All included studies were prospective in design. 19 21 22 23 24 Two studies evaluated focal cryoballoon ablation, 19 21 while three assessed multifocal cryoballoon ablation. 22 23 24 Across all studies, a total of 337 patients were included, of whom 69 (20.5%) underwent focal cryoballoon ablation and 268 (79.5%) underwent multifocal cryoballoon ablation. The included studies predominantly enrolled patients with short-segment BE.

Prior EMR had been performed in 160 patients, and 40 patients (12%) had undergone prior endoscopic ablation therapy. With respect to treatment protocols, three studies used a 10-second ablation duration, 21 23 24 one study used an 8-second duration, 22 and one study employed variable durations of 6, 8, or 10 seconds. 19

Baseline histopathology varied across studies: 1 patient (0.2%) had indefinite dysplasia, 9 (2.6%) had nondysplastic BE, 99 (29.4%) had LGD, 140 (41.5%) had high-grade dysplasia, 85 (25.2%) had intramucosal adenocarcinoma, and 3 (0.9%) had submucosal cancer. Detailed study and patient characteristics are presented in Table 1 .

**Table 1:** Baseline characteristics of included studies evaluating focal and multifocal cryoballoon ablation for Barrett’s esophagus.

Study	Frederiks 2026	Canto 2020	Canto 2018	Schölvinck 2014	Künzli 2016
Country	Netherlands, Germany, Belgium, France, Italy, and Ireland	USA	USA	Netherlands and the USA	Netherlands
Center	Multicenter	Multicenter	Single center	Multicenter	Multicenter
Study type	Prospective	Prospective	Prospective	Prospective	Prospective
Cryoballoon ablation type	Multifocal	Multifocal	Multifocal	Focal	Focal
TN (M/F)	107 (91/16)	120 (102/18)	41 (34/7)	39 (35/4)	30 (26/4)
Age	65 (10)	65 (45–83) ^#^	65.7 (34–79) ^#^	66 (57–69)*	66 (63–74)*
BMI	28 (5)	32 (18.7–59) ^#^	NI	NI	28 (21–35)*
Circumferential BE length (Prague C)	0 (0–1)*	1.2 (0–5) ^#^	1.7 (0–9) ^#^	2 (2–4)*	2 (1–4)*
Maximum BE length (Prague M)	2 (1–3)*	3.2 (1–6) ^#^	3.9 (1–14) ^#^	5 (3–7)*	0 (0–0)*
Preablation histology (LGD/HGD/IMA)	(34/34/36)	(29/67/24)	(13/23/5)	(9/9/11)	(14/7/9)
Prior EMR	64 (60%)	55 (46%)	14 (34%)	12 (31%)	15 (50%)
Prior RFA	0 (0%)	0 (0%)	19 (46%)	0 (0%)	23 (77%)
Preexisting esophageal stricture	NI	9 (8%)	9 (22%)	NI	NI
Number of ablation sessions	2 (2–3)*	2 (2–3)*	3 (2–4)*	1 or 2	1 or 2
Time of ablation	8 seconds	10 seconds	10 seconds	6/8/10 seconds	10 seconds
Follow-up biopsy protocols	Seattle protocol	Seattle protocol	Seattle protocol	NI	NI
Follow-up months	18 (9–26)*	12	21 (17.5–24.6)*	NI	NI

Data are presented as mean (standard deviation), median (range/interquartile range), or number (%), as reported in the original studies. BE, Barrett’s esophagus; BMI, body mass index; EMR, endoscopic mucosal resection; HGD, high-grade dysplasia; IMA, intramucosal adenocarcinoma; LGD, low-grade dysplasia; NI, not reported; RFA, radiofrequency ablation; TN, total number.

*Median IQR.

^#^ Mean range.

The overall risk of bias was judged to be serious across all studies, primarily due to the inherent risk of confounding associated with prospective, single-arm, nonrandomized study designs. Risk of bias in the remaining domains was generally low ( **Supplementary Table S3** ).

### Clinical Outcomes

CE-IM and CE-D were reported in all studies evaluating multifocal cryoballoon ablation. The pooled CE-IM rate by intention-to-treat analysis was 84% (95% CI, 72–91%; *I* ^2^ = 84.5%), and the pooled CE-D rate was 90% (95% CI, 76–96%; *I* ^2^ = 88%) ( Fig. 2 ). Sensitivity analyses demonstrated improved outcomes with reduced heterogeneity. The pooled CE-IM rate increased to 89% (95% CI, 83–93%; *I* ^2^ = 0%), and the pooled CE-D rate increased to 95% (95% CI, 90–97%; *I* ^2^ = 0%) ( **Supplementary Figures S1 and S2** ).

**Fig. 2:**
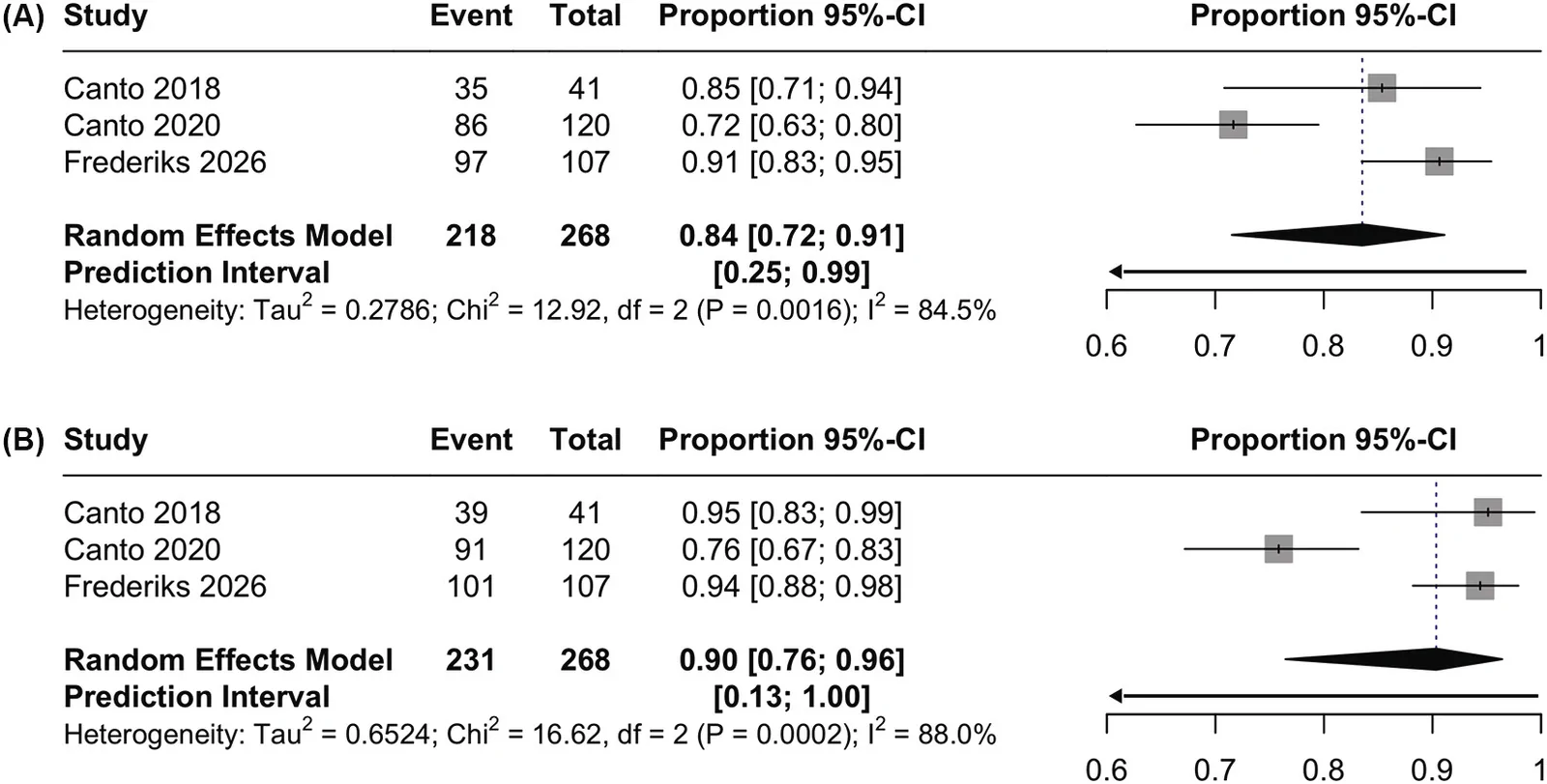
Forest plots of intention-to-treat eradication outcomes following multifocal cryoballoon ablation for Barrett’s esophagus. ( **A** ) Complete eradication of dysplasia (CE-D). ( **B** ) Complete eradication of intestinal metaplasia (CE-IM). Pooled estimates were calculated using a random-effects model with corresponding 95% confidence intervals.

In per-protocol analyses, the pooled CE-IM rate increased to 92% (95% CI, 87–96%; *I* ^2^ = 55.1%), and the pooled CE-D rate was 98% (95% CI, 94–99%; *I* ^2^ = 0%) ( Fig. 3 ). Sensitivity analyses using a leave-one-out approach demonstrated reduced heterogeneity with minimal impact on effect estimates; the pooled CE-IM rate was 90% (95% CI, 83–94%; *I* ^2^ = 11.2%). The pooled CE-D rate ranged from 96% to 99%, with no heterogeneity ( *I* ^2^ = 0%) ( **Supplementary Figures S3 and S4** ).

**Fig. 3:**
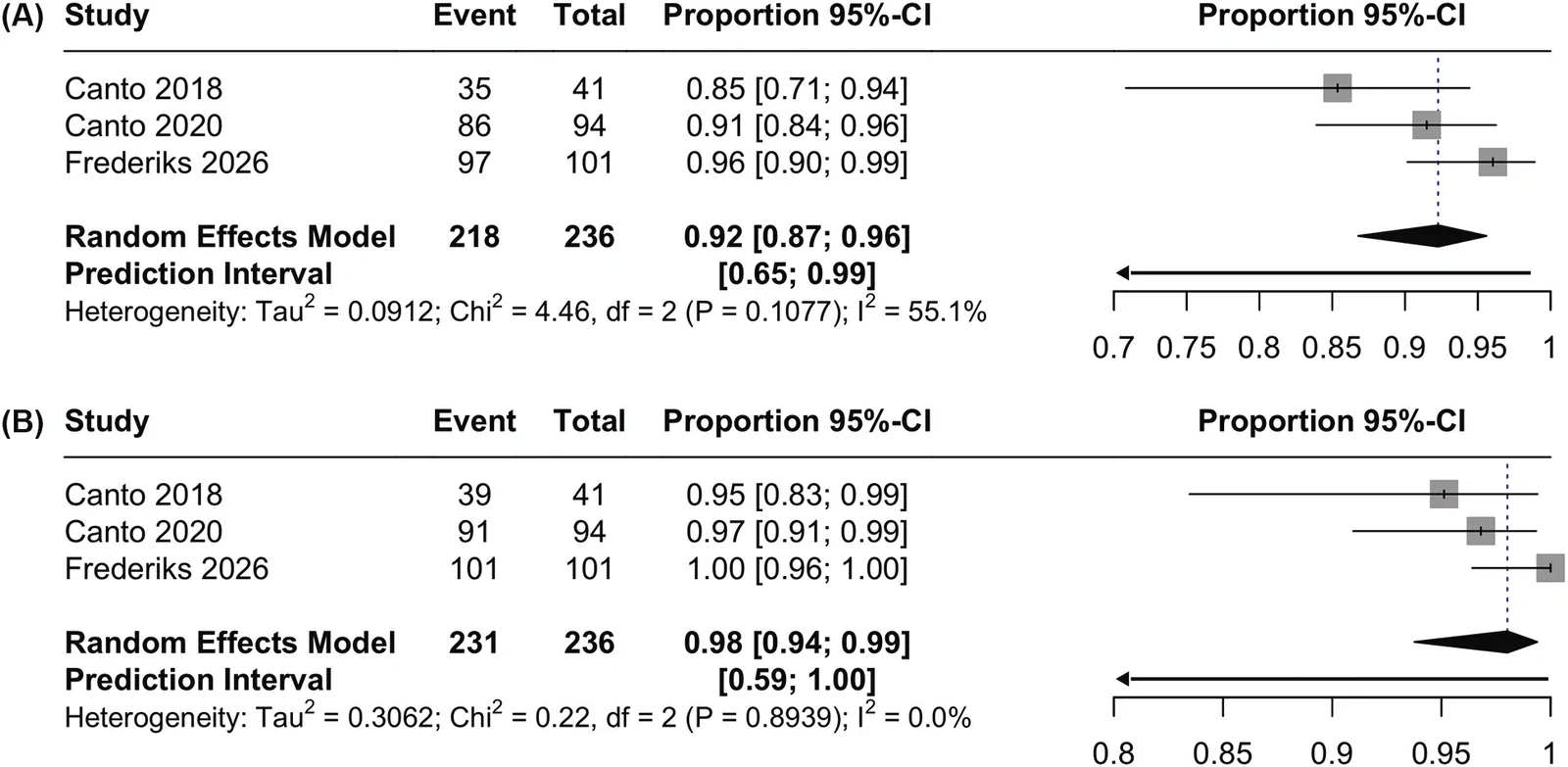
Forest plots of per-protocol eradication outcomes following multifocal cryoballoon ablation for Barrett’s esophagus. ( **A** ) Complete eradication of dysplasia (CE-D). ( **B** ) Complete eradication of intestinal metaplasia (CE-IM). Pooled estimates were calculated using a random-effects model with corresponding 95% confidence intervals.

In a subgroup analysis restricted to patients without prior endoscopic ablation, the intention-to-treat CE-IM rate was 84% (95% CI, 70–92%; *I* ^2^ = 84%), and the CE-D rate was 90% (95% CI, 75–96%; *I* ^2^ = 86.5%) ( **Supplementary Figures S5 and S6** ). Sensitivity analyses showed improved outcomes with elimination of heterogeneity; the pooled CE-IM rate increased to 90% (95% CI, 83–94%; *I* ^2^ = 0%), and the pooled CE-D rate increased to 95% (95% CI, 89–97%; *I* ^2^ = 0%) ( **Supplementary Figures S7 and S8** ).

Corresponding per-protocol rates were 93% (95% CI, 89–96%; *I* ^2^ = 38%) for CE-IM and 98% (95% CI, 94–100%; *I* ^2^ = 0%) for CE-D ( **Supplementary Figures S9 and S10** ). Leave-one-out sensitivity analyses demonstrated stable estimates with reduced heterogeneity; the pooled CE-IM rate ranged from 90% to 94% with no heterogeneity ( *I* ^2^ = 0%), and the pooled CE-D rate ranged from 97% to 99%, also with no heterogeneity ( *I* ^2^ = 0%) ( **Supplementary Figures S11 and S12** ).

### Technical Outcomes

The pooled technical success rate for multifocal cryoballoon ablation was 97% (95% CI, 95–98%; *I* ^2^ = 17.2%), compared with 92% (95% CI, 85–96%; *I* ^2^ = 0%) for focal cryoballoon ablation. The overall pooled technical success rate was 96% (95% CI, 94–98%; *I* ^2^ = 52.5%) ( Fig. 4A ).

**Fig. 4:**
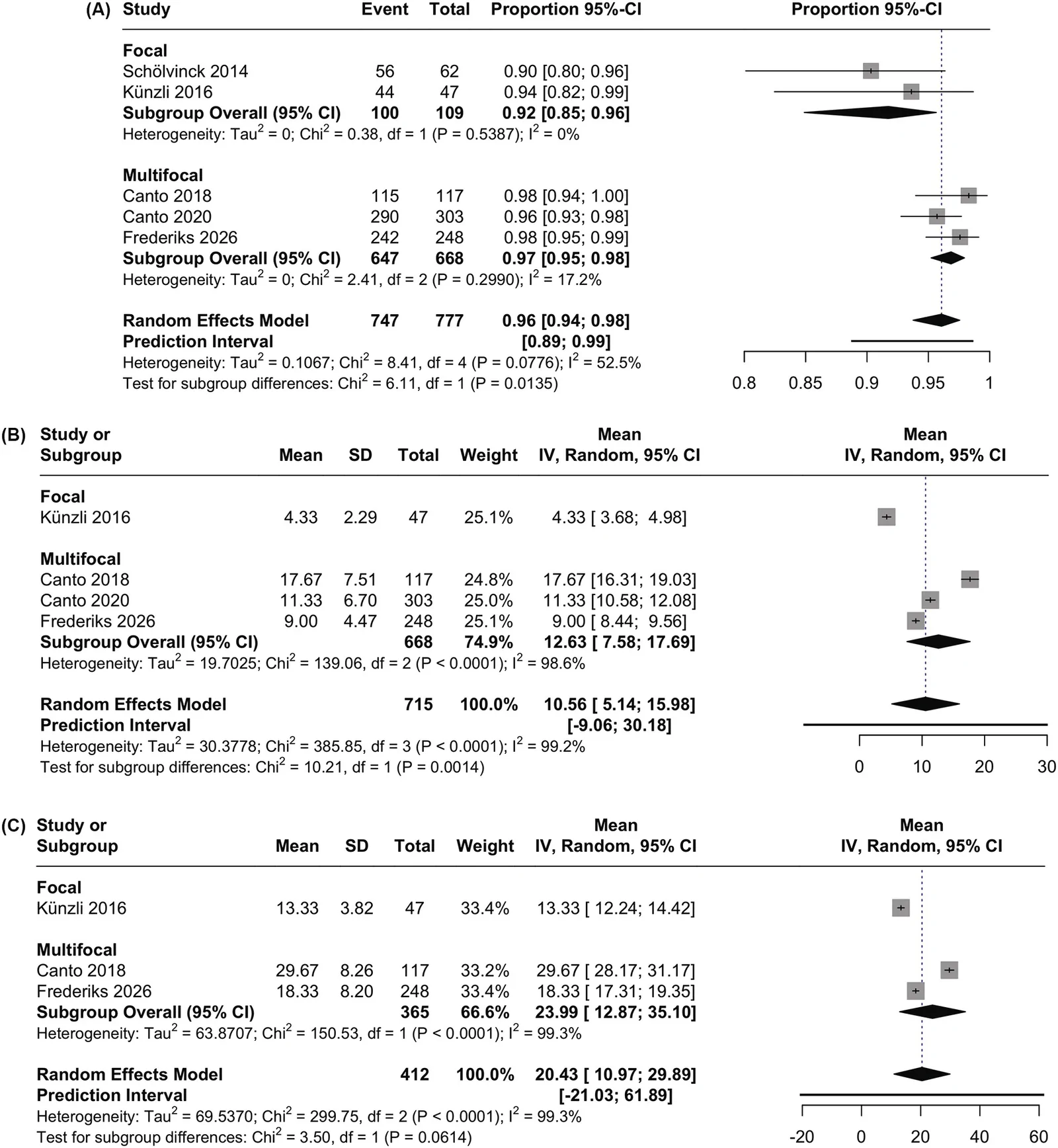
Forest plots of technical outcomes following focal and multifocal cryoballoon ablation for Barrett’s esophagus. ( **A** ) Technical success rate. ( **B** ) Mean ablation time. ( **C** ) Mean total procedure time. Subgroup analyses were performed according to focal versus multifocal cryoballoon ablation. Pooled estimates were calculated using a random-effects model with 95% confidence intervals.

The mean ablation time for multifocal cryoballoon ablation was 12.63 minutes (95% CI, 7.58–17.69; *I* ^2^ = 98.6%), whereas the overall mean ablation time was 10.56 minutes (95% CI, 5.14–15.98; *I* ^2^ = 99.2%) ( Fig. 4B ).

The mean procedure time for multifocal cryoballoon ablation was 23.99 minutes (95% CI, 12.87–35.10; *I* ^2^ = 99.3%), whereas the overall mean procedure time was 20.43 minutes (95% CI, 10.97–28.89; *I* ^2^ = 99.3%) ( Fig. 4C ).

### Safety

Esophageal strictures occurred in 12% of patients undergoing multifocal cryoballoon ablation (95% CI, 9–16%; *I* ^2^ = 0%), whereas no strictures were reported following focal cryoballoon ablation (0%; 95% CI, 0–100%; *I* ^2^ = 0%). The overall pooled stricture rate was 6% (95% CI, 2–18%; *I* ^2^ = 0%) ( Fig. 5A ).

**Fig. 5:**
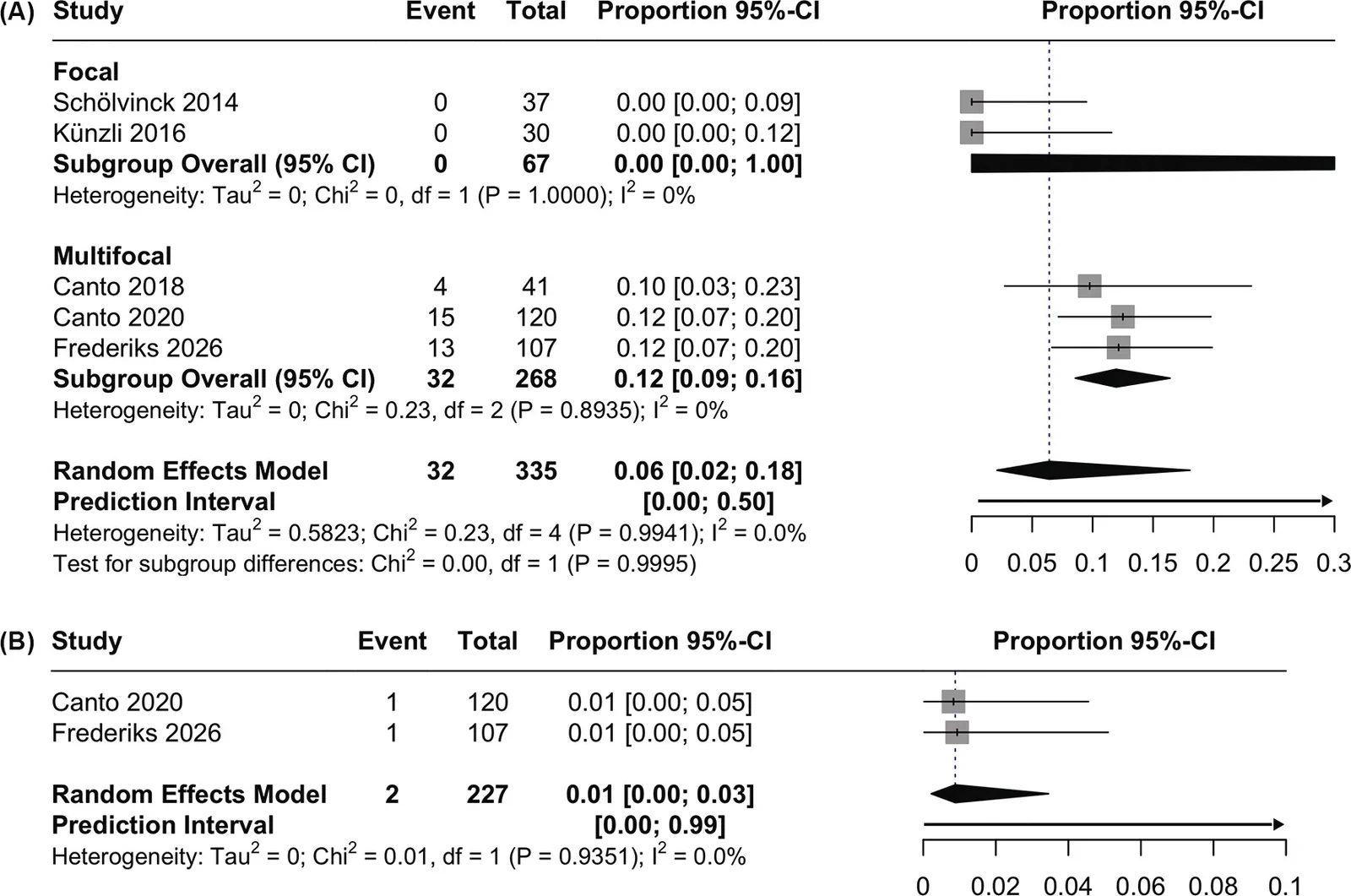
Forest plots of safety outcomes following focal and multifocal cryoballoon ablation for Barrett’s esophagus. ( **A** ) Esophageal stricture rates stratified by focal and multifocal cryoballoon ablation. ( **B** ) Incidence of buried Barrett’s glands following multifocal cryoballoon ablation. Pooled estimates were calculated using a random-effects model with corresponding 95% confidence intervals.

Buried Barrett’s glands were reported in two studies of multifocal cryoballoon ablation, with a pooled incidence of 1% (95% CI, 0–3%; *I* ^2^ = 0%) ( Fig. 5B ).

## Discussion

In this systematic review and meta-analysis of five prospective studies involving 337 patients, cryoballoon ablation demonstrated high rates of eradication of IM and dysplasia, along with a favorable safety profile, for the treatment of BE. The pooled rates of CE-IM and CE-D were 84% and 90%, respectively, in intention-to-treat analyses, rising to 92% and 98%, respectively, in per-protocol analyses. Technical success was high (96%), while the overall stricture rate was relatively low (6%), although it was higher in the multifocal subgroup.

The efficacy outcomes observed in this analysis are comparable to those reported for established ablative modalities, particularly RFA. Multifocal cryoballoon ablation achieved consistently high CE-IM and CE-D rates across both intention-to-treat and per-protocol analyses. Notably, these outcomes remained robust in patients without prior endoscopic ablation, supporting the role of cryoballoon ablation as a primary treatment modality. These findings are consistent with prior prospective studies reporting, by intention-to-treat, CE-IM rates ranging from 72% to 91% and CE-D rates ranging from 76% to 94%, and, by per-protocol, CE-IM rates ranging from 88% to 96% and CE-D rates ranging from 95% to 100%. 22 23 24

The heterogeneity observed in some ITT analyses likely reflects multiple factors, including differences in follow-up completion, patient selection, prior EMR status, Barrett’s segment length, and treatment protocols across studies. Notably, heterogeneity was markedly reduced in several PP and sensitivity analyses, suggesting that variations in follow-up and outcome ascertainment may have contributed to the observed between-study variability.

Comparative data further support these findings. Retrospective analyses have demonstrated similar short-term BE regression rates between cryoballoon ablation and RFA, 25 as well as comparable durability in maintaining remission of IM and dysplasia. 26 In addition, Canto et al. 2025 reported that, among patients achieving CE-IM, multifocal cryoballoon ablation was safe, effective, and durable for at least 3 years, with recurrence rates of IM and dysplasia of 2.9% and 0.99% per person-year, respectively. 32 Collectively, these data suggest that cryoballoon ablation is a viable alternative to RFA, with comparable efficacy in achieving and sustaining eradication.

Beyond RFA, additional ablative strategies are emerging. Hybrid APC, which combines submucosal fluid injection with thermal ablation, has demonstrated high eradication rates and a favorable safety profile in recent prospective multicenter studies and is increasingly considered, particularly for short-segment BE. 33

Technical success was achieved in the vast majority of procedures, with a pooled rate of 96%. Multifocal cryoballoon ablation demonstrated slightly higher technical success compared with focal ablation (97% vs. 92%), likely reflecting improved coverage of longer BE segments and refinements in device application. These findings are consistent with prior reports indicating technical success rates exceeding 95% for multifocal approaches 22 23 24 and slightly lower, though still more than 90%, rates for focal applications. 19 21

From a safety perspective, the overall stricture rate of 6% appears lower than that reported for RFA (7–15%), 4 5 6 7 although this may mask clinically relevant heterogeneity. Multifocal ablation was associated with a stricture rate of 12% (95% CI, 9–16%), a notable finding given the predominance of short-segment, noncircumferential BE and likely reflecting prior EMR-related fibrosis. As RFA cohorts often include longer, circumferential segments with inherently higher stricture risk, direct comparisons should be interpreted cautiously. In this context, the apparent safety advantage of cryoballoon ablation may be less pronounced than suggested by overall estimates.

Consistent with these observations, van Munster et al. reported lower postprocedural pain and reduced analgesic requirements with cryoballoon ablation compared with RFA; no strictures occurred in the cryoballoon group (0/20) versus 2 of 26 in the RFA group. 25 However, direct comparative data remain limited to small retrospective or nonrandomized studies.

The incidence of buried Barrett’s glands was low (1%), consistent with prior prospective data, suggesting that cryoballoon ablation does not appear to increase the risk of subsquamous IM.

An important procedural consideration relates to ablation duration. Earlier studies commonly used 10-second applications; however, emerging evidence suggests that shorter durations may reduce adverse events without compromising efficacy. Retrospective data indicate that 8-second ablation protocols achieve similar BE regression rates compared with 10-second applications, while potentially lowering the risk and severity of strictures. 34 These findings support considering shorter ablation durations as the standard approach, although prospective comparative studies are needed to define optimal treatment parameters.

This study has several limitations. The number of included studies was small, and all were prospective, single-arm, nonrandomized. Consequently, the overall risk of bias was judged serious due to the inherent confounding and the absence of comparator groups. Substantial heterogeneity across outcomes likely reflects differences in patient populations, BE segment length, prior treatments, and procedural protocols. Notably, a large proportion of patients had prior EMR and short-segment, predominantly noncircumferential BE; accordingly, the findings primarily pertain to postresection residual disease and may not be generalizable to treatment-naïve or longer, circumferential BE. Outcomes stratified by prior EMR status were not consistently reported, precluding subgroup analysis.

In addition, long-term durability data are limited, and direct comparative trials with other ablative modalities are lacking. Certainty of evidence was not formally assessed because the available evidence consisted exclusively of single-arm, noncomparative studies, and publication bias and meta-regression could not be reliably evaluated due to only five studies meeting the inclusion criteria.

To conclude, cryoballoon ablation demonstrates promising efficacy for achieving CE-IM and CE-D and appears to be associated with a favorable safety profile in the treatment of BE. Specifically, cryoballoon is a reasonable option for short-segment BE, especially post-EMR. However, these findings are derived from a limited number of nonrandomized studies with substantial heterogeneity, which constrains the strength of the inference. Larger, rigorously designed randomized controlled trials with extended follow-up are needed to more definitively determine its comparative effectiveness and safety relative to established modalities, including RFA.

## Data Availability

The data underlying this article are available within the article and its supplementary materials. Additional data are available from the corresponding author upon reasonable request.

## Supplementary Material

Ergänzendes Material Supplementary Material
